# Tinea Corporis Gladiatorum Presenting as a Majocchi Granuloma

**DOI:** 10.5402/2011/767589

**Published:** 2011-04-12

**Authors:** Anil Kurian, Richard M. Haber

**Affiliations:** ^1^Department of Medicine, McMaster University, 1280 Main Street West, Hamilton, Ontario, Canada L8S 4L8; ^2^Division of Dermatology, Richmond Road Diagnostic and Treatment Centre, University of Calgary, Rm 1153 1820 Richmond Road SW, Calgary, AL, Canada T2T 5C7

## Abstract

*Background*. Wrestlers are at increased risk of developing cutaneous infections, including fungal infections caused by dermatophytes. Erythematous lesions due to tinea infections can be mistakenly diagnosed as an inflammatory dermatitis and incorrectly treated with potent topical corticosteroid treatments which cause localized skin immunosuppression. This can eventuate in a Majocchi granuloma which then becomes refractory to topical antifungal therapy. To our knowledge, this is the first case of tinea corporis gladiatorum presenting as a Majocchi granuloma. *Observations*. A 20-year-old wrestler presented with a 4-year history of a large pruritic, scaly erythematous plaque with follicular papules, and pustules on his right forearm. The lesion had the clinical appearance of a Majocchi granuloma. He had been treated with potent topical corticosteroids and topical antifungal therapy. KOH and fungal culture of the lesion were negative. An erythematous scaly lesion in the scalp was cultured and grew Trichophyton tonsurans. Oral Terbinafine therapy was initiated and complete resolution of both lesions occurred within 6 weeks. *Conclusion*. The purpose of this report is to inform dermatologists that tinea corporis gladiatorum can present as a Majocchi granuloma and needs to be considered in the differential diagnosis of persistent skin lesions in wrestlers.

## 1. Case Report

**Figure 1:**
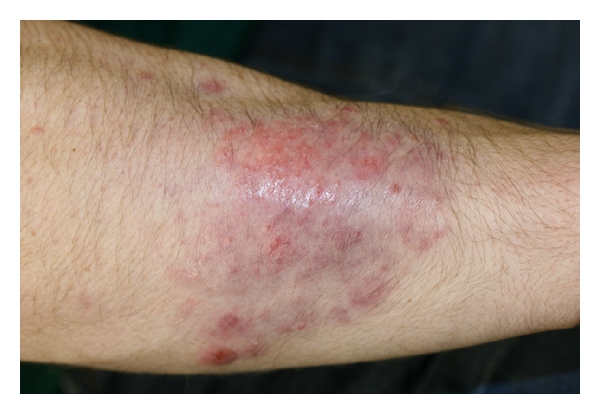
Clinical appearance of Majocchi granuloma.

**Figure 2:**
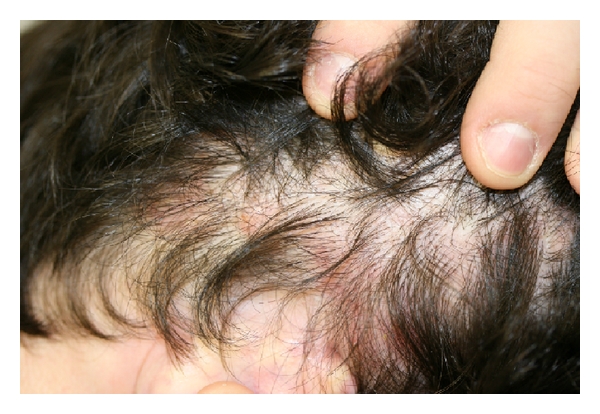
Clinical appearance of tinea capitis which grew *T. tonsurans*.

A 20-year-old man who was a high school and university wrestler for the past 6 years, presented with a 4-year history of a pruritic large scaly erythematous plaque with follicular papules and pustules on his right forearm (Figure 1). This lesion had the typical clinical appearance of a Majocchi granuloma. He had previously been treated with betamethasone dipropionate cream, diflucortolone valerate oily cream, fusidic acid cream, and terbinafine cream with no improvement. He also had several erythematous scaly patches with overlying alopecia in his right anterior scalp (Figure 2). KOH and fungal culture from the right forearm lesion were negative. A skin biopsy from the right forearm showed an acute deep folliculitis compatible with a Majocchi granuloma, but fungal stainings with a Grocott stain was negative. A skin biopsy was also obtained from the right forearm lesion for fungal and bacterial cultures. Fungal culture from the skin biopsy specimen revealed no fungal growth after 4 weeks incubation. Gram stain and bacterial culture were both negative as well.

**Figure 3:**
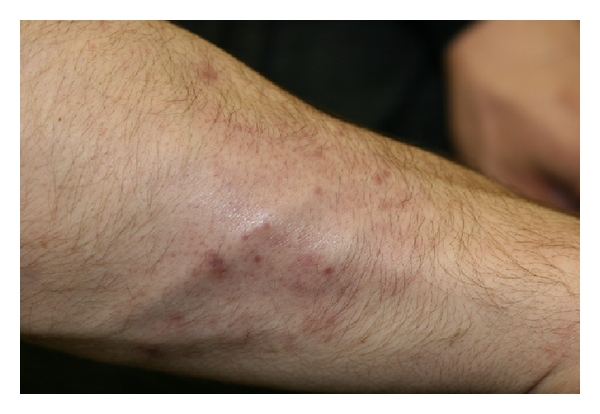
Resolution of Majocchi granuloma after oral Terbinafine therapy.

The diagnosis was finally established when a fungal culture from his scalp grew Trichophyton tonsurans. All topical therapy was stopped, and he was treated with oral Terbinafine 250 mg daily with marked improvement of his forearm (Figure 3) and scalp lesions in 2 weeks and complete clearing of both in 6 weeks.

## 2. Discussion

Cutaneous infections are relatively common among wrestlers due to close contact between opponents and the large percentage of abrasions involved in the sport. The most likely infections seen are of bacterial, viral, and fungal origin. Bacterial infections, such as cellulitis and impetigo, are frequently seen among adolescent wrestlers [1]. Viral infections, such as herpes gladiatorum caused by herpes simplex infections, may also be seen among 2–8% of amateur wrestlers [1]. These lesions present initially as stinging, painful eruptions with subsequent vesicular formation. Oral herpes simplex antiviral drugs should be started early to reduce the symptoms associated with active lesions and to prevent spread. Tinea infections are a common occurrence amongst all athletes, especially wrestlers, and most typically present with erythema, scaling, and pruritus. Individual studies have shown that as many as 75% of high school wrestling teams had tinea infections concurrently [2]. Topical antifungal treatment is first-line therapy for localized, discrete lesions, and oral antifungal therapies are used for multiple, widespread lesions. 

Fungal infections, such as tinea corporis, are commonly seen in wrestlers. *Trichophyton tonsurans *and *Trichophyton rubrum *are common dermatophytes implicated in tinea corporis. Tinea gladiatorum refers to transmission of a dermatophyte infection from close skin-to-skin contact of athletes. Among wrestlers, tinea corporis gladiatorum often presents as well-defined, erythematous, scaly plaques that manifest on the head, neck, and arms, which is a distribution consistent with the areas of skin-to-skin contact in wrestling [3]. It has been reported that *T. tonsurans *causes more outbreaks of tinea corporis in student wrestlers than *T. rubrum* [4]. Fungal infections can often disqualify or prevent a wrestler from competing in matches and therefore, rapid institution of therapy is necessary. Both topical and oral antifungal therapies have been proposed and used with success, however, the optimal therapeutic agent and its duration of use still remain uncertain at this time [3]. Regardless, antifungal therapy should not be delayed due to the contagiousness of the dermatophyte infection and also the impact that having a fungal infection can pose to a wrestler's competition status.

Tinea capitis is another commonly seen fungal infection in wrestlers. It is most frequently caused by the dermatophyte *T. tonsurans*. Surveys have shown that crowded living conditions, large family size, and low socioeconomic status may facilitate the spread of tinea capitis caused by *T. tonsurans* [5]. The clinical presentation of tinea capitis can vary from a scaly noninflamed dermatosis resembling seborrheic dermatitis to an inflammatory disease with scaly erythematous lesions and alopecia that may progress with deep inflammation leading to scarring and permanent alopecia. Treatment regimes should typically include oral Terbinafine or oral Itraconazole until complete eradication [6].

In addition to being the most frequent causative agent of tinea capitis, the anthropophilic organism *T. tonsurans *has also been implicated in the etiology of Majocchi granuloma. Majocchi granuloma typically occurs when a dermatophyte infection travels down a hair follicle in the setting of localized immunosuppression (most common a potent topical steroid) or systemic immunosuppression and leads to a suppurative folliculitis often producing a dermal granulomatous response. Immunosuppression allows the dermatophyte to penetrate deeper from its normal site in the stratum corneum, however, the patient's immune response to the fungus remains intact. It is mainly caused by *T. rubrum*, but there are a few reports of *T. tonsurans *being the implicated dermatophyte even in immunocompetent patients [7, 8]. Majocchi granuloma is clinically characterized by inflammatory papular, pustular, or nodular lesions mainly on the limbs or face. The trichophytin skin test is usually positive, implying an exposure to a superficial fungal infectious process, however, a clear histological dermatophyte infection is not always demonstrated in a Majocchi granuloma. In a prior study of four kerion cases and five cases of Majocchi granuloma, similar histological findings were observed in both conditions: a perifollicular infiltrate in 77.7% and fungal elements in 66.6% [9]. This study suggests that a diagnosis of Majocchi granuloma can still be made on clinical presentation without definitive fungal culture.

It would have been helpful to definitely establish a diagnosis of Majocchi granuloma in our case by fungal culture or by demonstrating fungal organisms in the hair follicles or dermis by fungal staining on the skin biopsy. Unfortunately, despite fungal skin scrapings and a skin biopsy cultured for fungus, a dermatophyte could not be isolated. Possibly previous treatment with a topical antifungal cream contributed to failure to culture or demonstrate fungal organisms from the forearm lesion. However, we feel this patient did have a Majocchi granuloma based on the clinical appearance with a chronic persistent erythematous scaly plaque with follicular papules and pustules, histology showing an acute deep folliculitis, negative bacterial culture of the biopsy from the forearm lesion, growth of *T. tonsurans* from the scalp lesion (the presumed cause of the Majocchi granuloma on the forearm) and the rapid response of both the forearm and scalp lesions to oral Terbinafine therapy.

We report the first case of tinea corporis gladiatorum presenting as a Majocchi granuloma in the medical literature. A literature search of Pubmed and Medline did not yield any other cases of Majocchi granuloma in patients with tinea gladiatorum. Clinicians should be aware that a Majocchi granuloma can occur in the setting of tinea corporis gladiatorum. Failure to consider the diagnosis could result in a significant delay in initiating proper therapy as was the case in our patient who went undiagnosed for 4 years.

## 3. Conclusion

Tinea corporis gladiatorum can present as a Majocchi granuloma, and we report the first case in the medical literature. Dermatologists must consider a Majocchi granuloma in the differential diagnosis of persistent skin lesions in wrestlers.
